# Reduction of Power Consumption by Chip Breakability Control in Ti6Al4V Titanium Alloy Turning

**DOI:** 10.3390/ma13112642

**Published:** 2020-06-10

**Authors:** Wojciech Zębala, Grzegorz Struzikiewicz, Bogdan Słodki

**Affiliations:** Production Engineering Institute, Cracow University of Technology, Al. Jana Pawła II 37, 31-864 Kraków, Poland; struzikiewicz@mech.pk.edu.pl (G.S.); bogdan.slodki@pk.edu.pl (B.S.)

**Keywords:** machining, turning, titanium alloys, chip breakability, power reduction, dry and high-pressure cooling

## Abstract

The paper concerns the problem of energy savings in turning of titanium alloy Ti6Al4V. Since this alloy belongs to difficult to cut materials, there is a problem with chip forming and breaking. The turning process is often supported by implementing a high-pressure cooling (HPC) system. Based on the observations and the adopted chip classification method, the authors proved that it is not necessary to use this method in roughing operations, however it helps with the chips breaking process in finishing operations. A general algorithm for machining optimization due to the chip geometry is presented and described. In the presented case, it was shown that the acceptable chip geometry could be obtained with a reduced power consumption by approximately *Pc* = 0.5 kW. The authors concluded that it was not necessary to apply cutting data and a coolant system to achieve perfect chip geometry. An acceptable form was often sufficient, while requiring less energy. An additional factor resulting from the operation of systems supporting the cutting process, such as an HPC device, should be taken into account in the formula concerning the energy consumption (EC) of a computerized numerical control (CNC) machine tool.

## 1. Introduction

World electricity consumption increased by 4% last year to over 23,000 TWh, and the share of electricity in global energy consumption was 20%. According to the data from IEA [1], the largest end user of energy is transport (36%) and manufacturing, which is responsible for 23%. Machining is one of the most commonly used and energy consuming manufacturing processes [2]. Therefore, research is being carried out and methods are being developed to optimize and reduce energy demand. In the case of machining, methods are mainly used to reduce the value of cutting forces, increase productivity and optimize processes. Bhaumik et al. [3] analyzed machining titanium alloys with tools made of more durable tool materials, in particular cubic boron nitride (CBN). Da Silva et al. [4] mentioned that the use of coatings for cutting tools reduces the coefficient of friction. Zhang et al. [5] described the use of new insert designs like Wiper. Many authors analyzed the issue of process optimization, due to the time, number and place of machining operations. This was described by Rao et al. [6], Słodki et al. [7] and Jawahir et al. [8]. Currently, due to the use of new machining methods, like Done in One [9], the optimization of tool movement trajectories to increase process efficiency is becoming increasingly important. In this context, Bohez et al. [10] described the optimization of tool motion kinematics.

Increasing productivity while reducing energy, material and tool costs is particularly difficult when machining difficult-to-cut materials [11], including titanium alloys [12,13]. These materials have found a wide application in many industrial branches, such as chemical, petrochemical and shipbuilding, because of their excellent corrosion resistance. Niinomi [14] and Sha et al. [15] analyzed the usage of titanium alloys in medicine and dental for surgical and prosthetic implants, as well as in the automotive, aviation and space industries in engine and chassis components, due to the high strength-to-weight ratio (titanium density is around 60%, compared to super alloys based on nickel or cobalt).

Improvement in the machinability of titanium alloys can be achieved by using various techniques from dry machining [16,17] to cooling and lubrication of the cutting zone [18] such as minimum quantity lubrication (MQL) machining [19,20], cryogenic machining or high-pressure Cooling (HPC) machining, i.e., the use of systems enabling feeding of cutting fluid to the cutting zone with increased pressure (from 50 to 355 bar, and even 1000 bar). It was analyzed by many authors, such as Ezugwu et al. [21], Çolak [22], Khan et al. [23] and Da Silva et al. [4].

A separate issue is the chip forming process, especially in machining difficult-to-cut materials, including titanium alloys. It has been the subject of analyzes undertaken by many researchers (e.g., [7,12,24,25]). In industrial practice, chip geometry and its varieties can take different forms, such as short and long ribbon, spiral, helical, arc, etc. [24,25]. The radius of curvature of the chip depends on the tool geometry and degree of wear, workpiece properties and cutting parameters, mainly feed [12]. Currently produced cutting inserts usually have rake face with a chip breaker or a chip former (name depends on a tool manufacturer). Chip breaking usually results from a chip hitting the raw surface of the workpiece, or the flank face of the insert. It has been shown [26] that, in the process of turning difficult-to-cut materials, the dominant way of chip breaking (for 85% of cases) is chip hitting the flank face.

The main area of research regarding machining of titanium alloys is the impact of cutting speed, feed and cutting depth on the quality of the machined surface, chip formation and breaking, determination of the value of cutting forces and temperature [27,28]. Palanisamy et al. [29] conducted research on turning of the Ti6Al4V alloy. A series of experiments were carried out at various cutting parameters and pressure levels (i.e., 6 bar—standard pressure—or 90 bar—high coolant pressure), for machining with uncoated cemented carbide inserts. Studies have shown that the use of a high-pressure technique improves chip breaking and evacuation, due to the mechanical effect of high-pressure fluid. They also found that HPC treatment increased tool life almost three times, compared to conventional cooling. Çolak [22] investigated the machinability of titanium alloy in conventional and high-pressure cooling conditions at different cutting speeds, feeds and depths of cut with a carbide tool coated with (Ti, Al) N + TiN. He stated that feeding the coolant under high pressure to the cutting zone reduces the values of the cutting forces, ensures the desired chip breaking and reduces wear of the cutting tool. Da Silva at al. [4] analyzed the tool wear mechanism during high speed machining of titanium alloys. Tool life decreased with increasing cutting speed, despite the cooling system. A meaningful improvement of productivity was obtained when machining with a high pressure coolant supply, which causes the lowering friction and temperature and influence on the reduction of the tool-chip contact length and chip curl radius [21,30]. Segmented chips were generated when machining with HPC system. In case of machining with conventional coolant flow continuous chips were forming. In turn, Kamiński et al. [28] analyzed the temperature in the cutting zone under HPC conditions. The authors say that the application of coolant pressure between 20 and 70 MPa decreases the temperature in conventional cooling in comparison with dry machining (about 15%). The reduction of temperature when HPC is used may be even about 40%–45%. Higher coolant pressure strongly influences the chips breaking.

Many researchers were involved in optimization of titanium alloys machining using various methods and mathematical models [31]. For example, Cus et al. [32] demonstrated the usefulness of genetic algorithms (GA) for online optimization of cutting parameters in the milling process. Heron et al. [19] and Wang et al. [33] developed an optimization model for the turning process, taking into consideration factors affecting machining, such as: cutting force (*F_c_*), tool life (*T*), surface roughness (*Ra*), material removal rate and chip breaking.

The purpose of the article is to develop a method for reducing power demand in turning Ti6Al4V titanium alloy using the HPC system. The paper consists of four sections. After introduction, the second section describes the experimental procedures and case study, and offers a description of chip breakability and the energy requirements of turning titanium alloy. Results of the experiments are presented in section three. At the end of the paper (Section 4), the authors summarize the investigation.

## 2. Materials and Methods

### 2.1. Experimental Procedures and Case Study

The tests were carried out for the case of turning the titanium alloy Ti6Al4V, dry and under increased coolant pressure (*p* = 70 bar). Table 1 and Table 2 present the mechanical properties and chemical composition, respectively.

Applied cutting data: cutting speed *v_c_* = const. = 50 m/min; depth of cut *a_p_* = 1.0 and 3.0 mm, feed rate *f* = 0.15; 0.20; 0.25; 0.30 mm/rev for turning without coolant and with a coolant pressure *p* = 70 bar. Cutting data were selected in accordance with the recommendations of the cutting tool manufacturer [34] (Sandvik Coromant, Sandviken, Sweden). Cutting tests were performed on a Mazak Integrex 200-IV CNC (Yamazaki Mazak Corporation, Takeda, Japan) machine tool with a Mazatrol Matrix control system. ToolWay S432 7% (Statoil Poland Sp. z o. o., Kraków, Poland) coolant was used.

**Figure 1:**
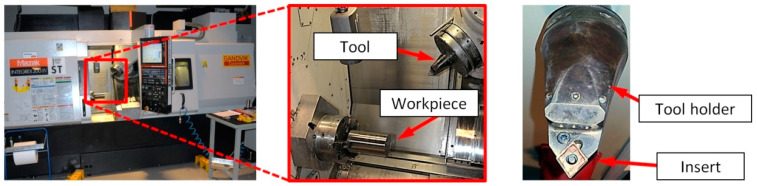
Test stand with the cutting tool.

Cutting inserts CNMG 120408-SMC (nose radius *r*_Ɛ_ = 0.8 mm) grade 1115 and tool holder C6-PCMNN-00115-12HP produced by Sandvik Coromant (Sandviken, Sweden) were applied in research. The test stand with the cutting tool is shown in Figure 1.

The first stage of experimental research was to analyze the impact of variable cutting parameters (feed *f* and cutting depth *a_p_*) and the cooling method on the values of components of the total cutting force. Additionally, the chips and machined surface was observed by the means of 3D Keyence microscope (Osaka, Japan).

During research, the chips form and cutting force components were measured. The Kistler (Winterthur, Switzerland) system was implemented for measurements of the cutting force components (dynamometer 9257B and amplifier 5070B). Taguchi’s method [35] was used to establish an experimental research plan. The influence of cutting parameters and pressure (*p*) on the components of the cutting force (*F_c_*) and (*F_f_*) and form of chips was analyzed.

In the statistical analysis of the test results, the model of the matching function was adopted according to Equation (1) [35].

(1) $$Y_{1}=y-\varepsilon =b_{0}x_{0}+b_{1}x_{1}+b_{2}x_{2}+b_{3}x_{3}+b_{4}x_{4},$$

where: *Y*_1_—the estimated response based on first order equation, *y*—the measured parameter (e.g., surface roughness) on a logarithmic scale, *x*_0_ = 1 (dummy variable), *x*_1_–*x*_4_—logarithmic transformations of cutting speed, feed rate, depth of cut and nose radius, respectively, *Ɛ*—the experimental error, *b*_0_–*b*_4_—the estimates of corresponding parameters.

The S/N ratio analysis strategy was adopted as “the lowest-the best”, according to Equation (2) [35].

(2) $$S/N=-10\cdot \log\left(\frac{1}{n}\overset{n}{\underset{i=1}{\sum }}y_{i}^{2}\right)$$

Table 3 presents the test plan together with the actual values of the cutting parameters used in research.

### 2.2. Chip Breakability Description

The control of the chip breaking process and the method of removing chips from the cutting zone is necessary for the correct course of the machining process on numerically controlled machine tools. Therefore, research was carried out on the control of chip geometry in the machining process, as presented by Ezugwu and Bonney [30]. The analysis of the influence of cutting parameters (feed, cutting depth, cutting speed) on chip control is presented by Fang et al. [36], and the procedure for predicting the shape of chips is described by Fei and Jawahir [37]. Lee et al. [38] used these procedures and their modifications in their works. The factors associated with the cutting tool can also affect the chip forming and breaking process. For example, the authors of [39] and Ren et al. [40] presented the results of tests that concerned the impact of chip breaker geometry or protective coating on the cutting insert rake face. In turn, Pusavec et al. [41] and Yi et al. [42] discussed issues related to the optimization of machining of difficult-to-cut materials on CNC machine tools, due to carbon emissions. From such a chip geometry description (chip groups, i.e., arc/bulky, spiral/circular) a chip can be described through the use of both linguistic and numerical information [36]. A detailed description of this method is provided in Pusavec et al. [41] and Fang et al. [36].

**Figure 2:**
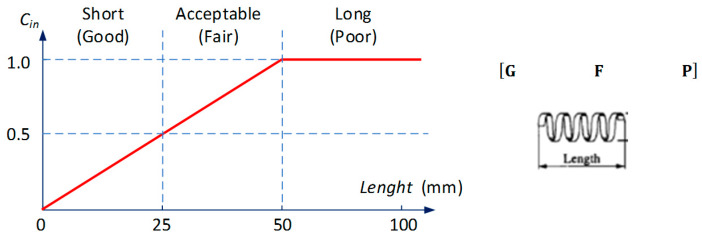
Simplified classification of chip geometry (due to the length of chip (*Lch*)).

Knowing the chip group, and dimensional features, the chip dimensional features can be converted to numeric value. It should be noted that, from a practical point of view (industrial applications), only the chip length value can be an indicator of the correct chip geometry (i.e., the longer the chip, the worse its form). Determining this value does not require specialized measuring devices, and it is easy to determine the dimensions. Therefore, the authors adopted a simplified chip classification method shown in Figure 2, based on Pusavec et al. [41].

Having characterized the chip dimensional features, chip has to be evaluated from the chip breakability point of view.

The actual chip can be ranked into three different grades (good, G, fair, F and poor, P), representing the level of chip breakability. The chip breakability index, *C_in_* (Equation (3)), is calculated in the range from 0 to 1. The classification has been adopted for the length of chips (*Lch*):

- *Lch* ≤ 25 mm—short chips, correct (breaking index 0 < *C_in_* <0.5);
- *Lch* > 25 mm and *Lch* ≤50 mm—fair, acceptable chips (fragility coefficient 0.5 < *C_in_* <1.0);
- *Lch* > 50 mm—long, unfavourable chips (brittleness coefficient *C_in_* = const. = 1.0). Lower *C_in_* values represent better chip breakability.

(3) $$C_{in}\left(L_{ch}\right)=\{\begin{array}{c} 0.02\cdot L_{ch}\;if\;0\leq L_{ch}\leq 50\;\\ 1\;if\;L_{ch}>50 \end{array}$$

### 2.3. Energy Requirement

**Figure 3:**
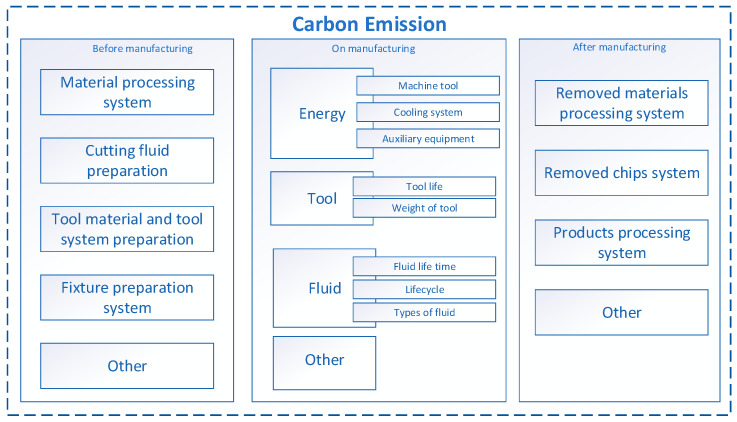
Components of carbon emission of CNC machining system.

Energy requirement (power consumption) is an important component in CNC machining systems. Actions aimed at reducing power consumption during machining directly affect lower carbon emissions into the environment [43]. Based on analysis of publications Gutowski et al. [2], Jia et al. [43] and Yi et al. [42] the scheme of carbon emissions of a CNC machining system was developed by the authors, and is shown in Figure 3.

Yi et al. [42] found that the carbon emissions of a CNC machining system (*CE*) are indirectly generated from the production of raw materials (*CE_m_*), cutting tools (*CE_tool_*) and cutting fluid (*CE_fluid_*), the electricity consumption (*CE_elec_*) and the fabrication of chip removal (*CE_chip_*). The total carbon emissions from the CNC manufacturing system (*CE_p_*), as shown in Equation (4) [42], are the sum of emissions from the stages before manufacturing (*CE_befor manufacturing_*), during manufacturing (*CE_on manufacturing_*) and after the part has been made on a machine tool (*CE_after manufacturing_*).

(4) $$CE_{p}=CE_{befor\;manufacturing}+CE_{on\;manufacturing}+CE_{after\;manufacturing}$$

However, the authors excluded *CE_chip_* and *CE_m_* components in their carbon emission model, due to their low impact. It should be noted, however, that meeting the requirements of the proper conduct of the machining process due to the form of chips can be burdened with an additional cost, i.e., energy consumption and an increase in the carbon expenditure into the environment. It results directly from the method of cooling the cutting zone. For example, the use of HPC or cryogenic treatment requires the use and manufacture of additional equipment in the production process, which results in increased power consumption and carbon expenditure (for example, the power of a device providing pressure up to 80 bar is 7.5 kW) (ChipBlaster Inc. Meadville, PA, USA [44]). In addition, carbon emissions increase during the process, due to the type and amount of liquid required (for example, the liquid output of devices used in industry is 20–80 L/min), and meeting the requirements for the purity of the cooling medium (e.g., a 20 micron filter is required) and an additional tank. All these factors also increase the cost of producing parts in the manufacturing process. The reduction of power demand in the process translates directly into carbon emissions (*CE_elec_*), described by the general formula [42]:

(5) $$CE_{elec}=Activity\times Emission\;Factor=EC_{process}\times CEF_{elec}$$

where *CE_elec_* is expressed as the emission factors of electricity *CEF_elec_* (kgCO_2_/kJ) and the total energy consumption of the whole process, *EC_process_* is the energy consumption of the CNC machine tool.

(6) $$EC_{process}=P_{air}+P_{c}+P_{a}$$

where:

*P_a_*—load loss power while machine tool is working;
*P_air_*—machine tool power consumption when idle;
*P_c_*—cutting power.

Patterns and description for air cutting power *P_air_* were presented by Shi et al. [45], cutting power *Pc* was described by Rajemi et al. [46]. In turn, load loss power *Pa* was explained by Liu et al. [47]. However, in the above relationship, the power of additional external systems embedded in the CNC machine tool and supporting the cutting process is not explicitly (separately) considered, which could be useful for machine tools that are not standard equipped with HPC system Therefore, according to the authors of this paper, the formula for energy consumption of the CNC machine tool (*EC_process_*) should take into account an additional factor, i.e., the power consumption resulting from the work of cutting process supporting systems (*P_add system_*), which is shown in Equation (7):

(7) $$EC_{process}=P_{air}+P_{c}+P_{a}+P_{add\;system}$$

**Figure 4:**
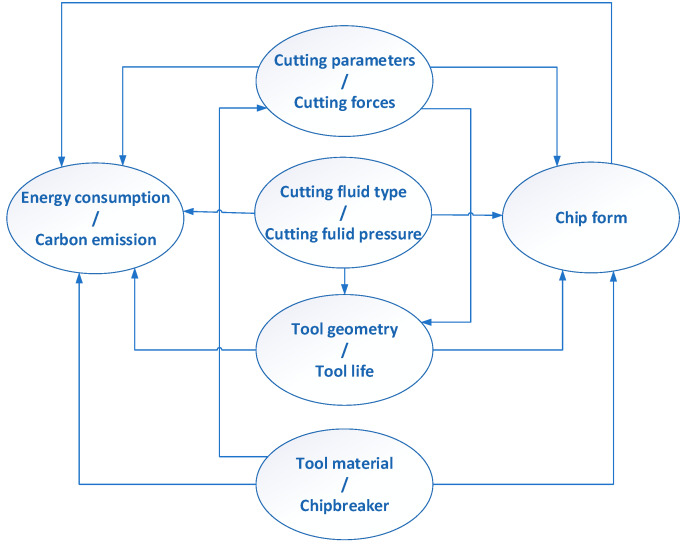
Influence and interdependence of cutting parameters on current consumption and chip geometry.

In the cutting process, a change of one parameter directly affects the change of other parameters describing the process. Figure 4 shows the correlation between cutting parameters and their effect on current consumption and chip geometry. For example, a change increasing the feed value leads to the correct chip geometry, but, at the same time, the change results in a decrease of the tool life and an increase of cutting forces, and, thus, an increase in current consumption and carbon emissions. Similarly, the shape of the chip breaker directly affects the form of the chips and the power demand, as well as the change in the value of cutting forces, which causes a change in the tool, etc.

The values of the cutting parameters, geometry and material of the tool, as well as the shape of the chip groove, are selected before the machining process. Therefore, chip control can be implemented by changing the cooling method (e.g., dry, MQL, Crio machining)—or by increasing the coolant pressure—in HPC machining. Companies producing cutting tools recommend chip breaker shapes and cutting parameter ranges, for which a correct chip geometry is obtained for dry machining or typical (flooding) cooling of the cutting zone. Therefore, the question arises about the synergy of cutting parameters, the shape of the chip breaker and the value of coolant pressure. The consequence of this is the question at which value of the coolant pressure, a correct chip geometry is received.

**Figure 5:**
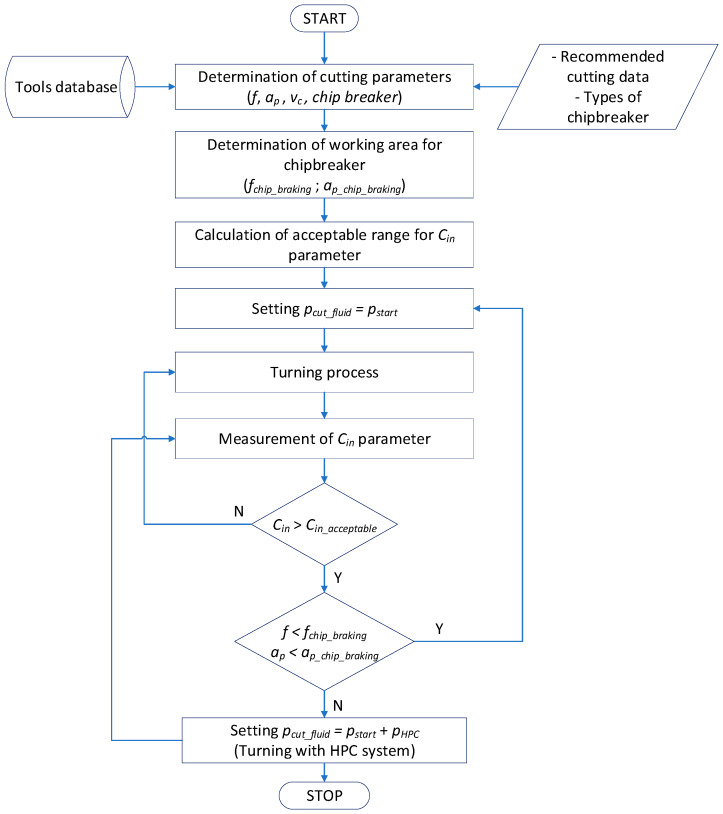
General algorithm for setting the cutting parameters and coolant method because of the chip geometry.

Figure 5 shows a general algorithm for minimizing power consumption in the turning process using HPC system, due to the chip geometry. The algorithm takes into account the cutting tool ranges (depth and feed) recommended by the manufacturer, for which a correct (broken) chip geometry and type of cooling (dry and HPC) are obtained. Based on the cutting tool manufacturer’s recommendations, the cutting parameters (*f*, *a_p_*, *v_c_*) and the chip breaker type are adopted. Next, the chip breaker working area is determined, i.e., the parameter ranges for *f_chip breaker_* and *a_p chip breaker_*. The chip geometry is determined by the chip breakage index *C_in_*. The turning process starts with dry machining. Then, during the cutting process, the condition of exceeding the accepted value of the *C_in acceptable_* index is checked, and the area of the chip breaker working field is used for the feed *f* and cutting depth *a_p_*. In the next step, if the chips are obtained in an unfavorable form (*C_in_* > *C_in acceptable_*) and the incorrect operation of the chip breaker, the decision is made to apply increased coolant pressure. In the case where the chip breaker performs its work correctly (short chips are obtained), HPC machining is not applied.

## 3. Results of Experiment and Discussion

The influence of the variable cutting parameters on the values of the total cutting force components, main *F_c_*, feed *F_f_* and radial *F_p_*, was analyzed. Table 4 presents the results of the S/N parameter and the average values of the individual components obtained in the individual tests system.

**Figure 6:**
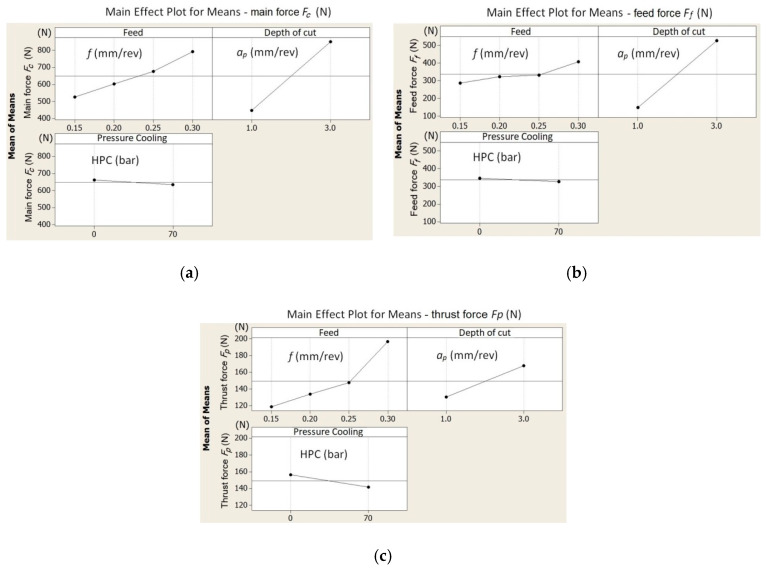
The influence of the cutting data on the values of the total cutting force components: (**a**) *F_c_*, (**b**) *F_f_*, and (**c**) *F_p_*.

Figure 6 shows, in graphic form, the influence of the particular cutting data on the values of the cutting force components.

Analysis of the test results showed that the coolant pressure value has no significant effect on the values of the components of the total cutting force. An increase of the pressure to 70 bar caused a decrease of the values of *F_c_* and *F_f_* by several percent (3%–8%). A greater pressure effect (9%–15%) is visible for the *F_p_* component (Figure 6c). It may result from the direct influence of the cutting fluid stream fed in the same direction, i.e., the addition of forces resulting from the machining process and the pressure of the liquid.

Table 5, Table 6 and Table 7 show the analysis of variance (ANOVA) regression analysis results of the components for the total cutting force (where: *DF*—degrees of freedom, *Seq SS*—sums of squares, *Adj SS*—adjusted sums of squares, *Adj MS*—adjusted means squares).

**Figure 7:**
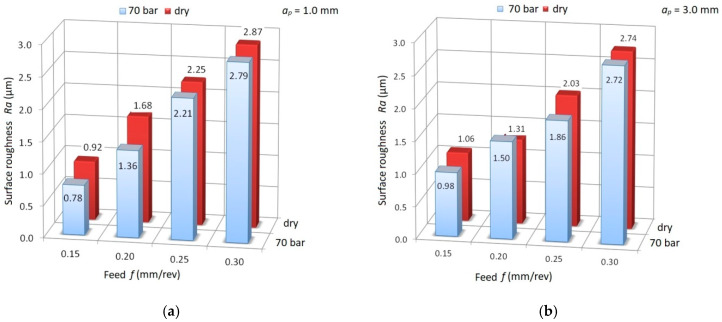
Dependence of surface roughness (*Ra*) for dry and high-pressure cooling (HPC) turning for (**a**) cutting depth *a_p_* = 1.0 mm; (**b**) cutting depth *a_p_* = 3.0 mm.

The parameters significantly affecting the values of the cutting forces are the feed and depth of cut. An increase of the value of the peripheral component *F_c_* by about 200 N was observed when changing the feed from *f* = 0.15 mm/rev to *f* = 0.30 mm/rev. A similar trend is visible for changing the depth of cut. The research also showed no significant effect of coolant pressure on the surface roughness values *Ra*. Figure 7 shows a comparison of the measurement results for *Ra* for dry machining and HPC.

**Figure 8:**
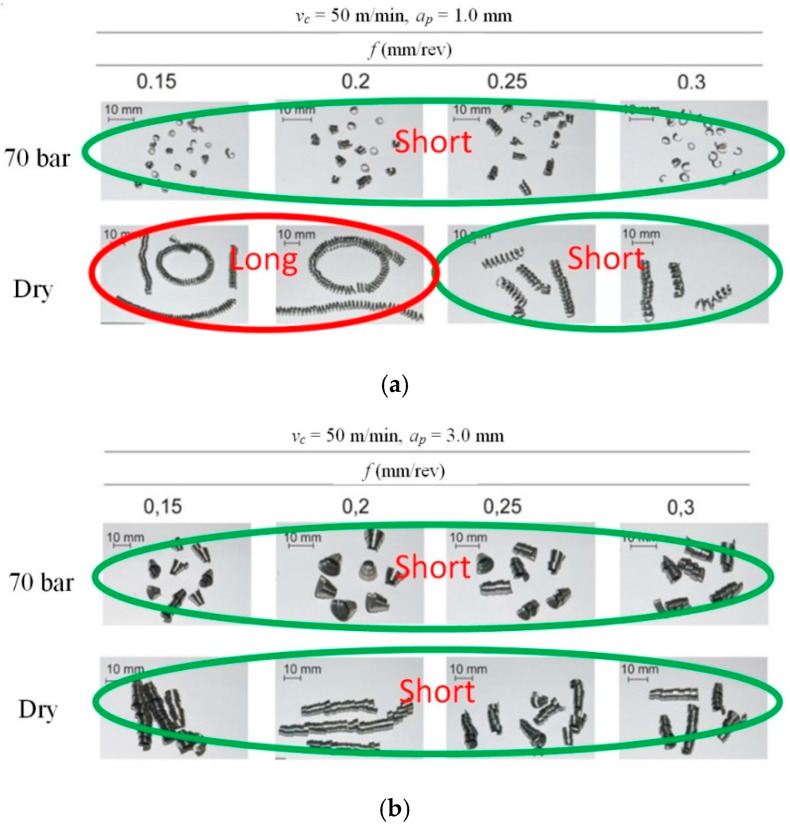
Chip geometry classification for HPC machining and dry machining as a function of the feed for (**a**) cutting depth *a_p_* = 1.0 mm; (**b**) cutting depth *a_p_* = 3.0 mm.

It follows from the above that increasing coolant pressure in the cutting process mainly supports the chip breaking process. Therefore, an analysis of the chip geometry obtained in experimental studies was carried out. Figure 8 shows photographs of chips of various forms. Short, broken chips were obtained in all cutting tests with HPC pressure *p* = 70 bar. For dry machining, a change of the form of chips from long to short was observed by increasing the feed values *f* and cutting depth *a_p_*, and, hence, higher values of the cross-section of the chip were created. This is due to the better filling of the chip groove by the created chip. This phenomenon was also analyzed by Balaji et al. [48].

The next step taken in the research was to determine the effect of cutting zone cooling on the power consumption (*Pc*) and the chip breaking index (*C_in_*). Power value was calculated according to the formula:

(8) $$P_{c}=\frac{F_{c}\times v_{c}}{60}(W)$$

In Table 8 and Table 9, the results of measurements of the average chip length (*Lch*), the power (*Pc*) and the chip breaking coefficient (*C_in_*) are compared.

**Figure 9:**
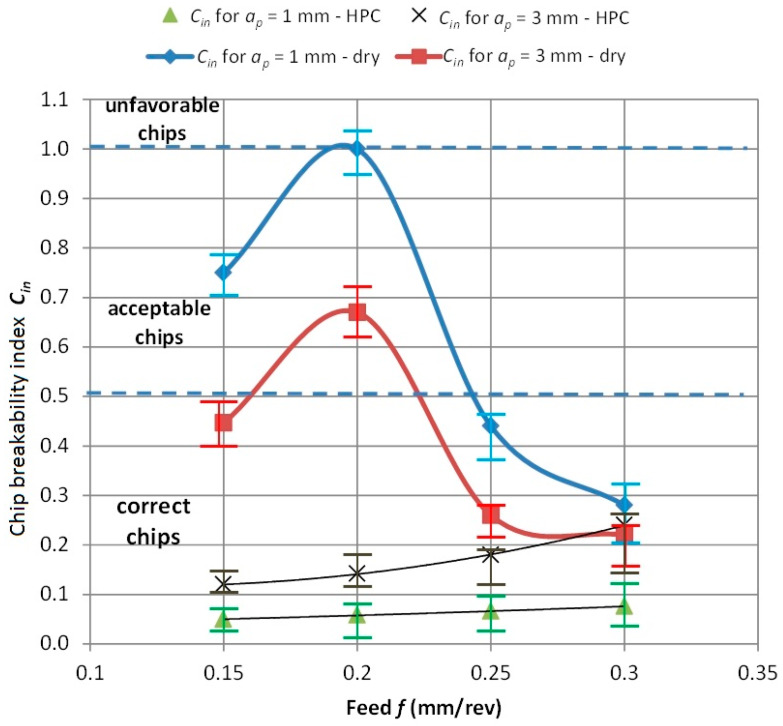
Relationship between the chip breaking index (*C_in_*) and the feed (*f*).

**Figure 10:**
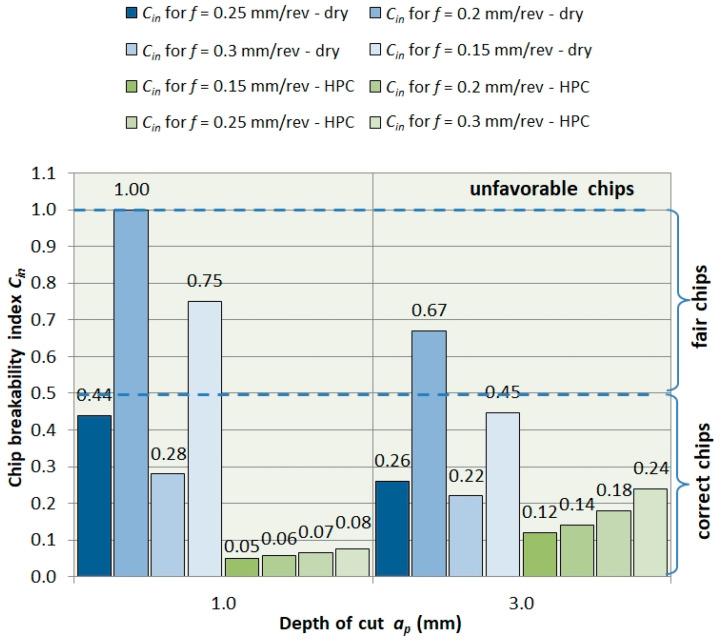
Dependence of the chip breaking index (*C_in_*) from the cutting depth *a_p_*.

Figure 9 and Figure 10 show the dependence of the chip breaking index *C_in_* from the feed *f* and depth of cut *a_p_*. The drawings indicate three areas for classification of correct, acceptable and unfavorable chips. It can be seen that in the case of dry machining, the chip geometry is variable and depends on the feed value *f*. In the feed range of 0.15 < *f* < 0.25 mm/rev, three forms are observed. For tested feed *f >* 0.25 mm/rev, a correct chip shape was obtained with the *C_in_* coefficient similar to HPC machining.

In turn, increasing the depth of cut *a_p_* strengthens the chip breaking process Figure 10. Chip geometry tends towards correct chips. For the HPC turning case, in all analyzed cases, the chips had a correct form.

**Figure 11:**
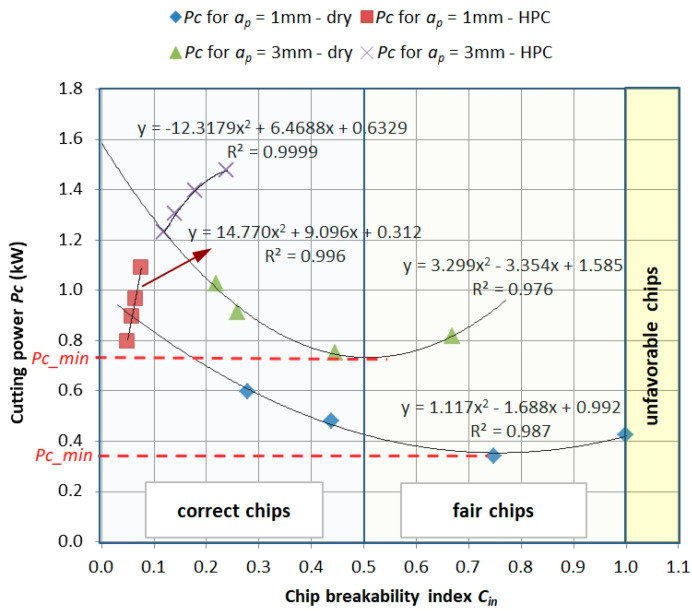
Power demand (*Pc*) as a function of chip breaking coefficient (*C_in_*).

Figure 11 shows the dependence of the *Pc* power demand as a function of the chip breaking ratio *C_in_*. The presented relationships show that a correct and acceptable form of chips can be obtained with a reduced demand of cutting power. The minimum value *Pc_min_* depends on the feed value *f* and depth of cut *a_p_*, i.e., on the cross-section of the cut layer. The machining method, in this case the change from dry machining to HPC machining, reduces the value of the *C_in_* chip breakage index (i.e., shorter, more broken chips) but also significantly increases the demand for cutting power *Pc*.

**Figure 12:**
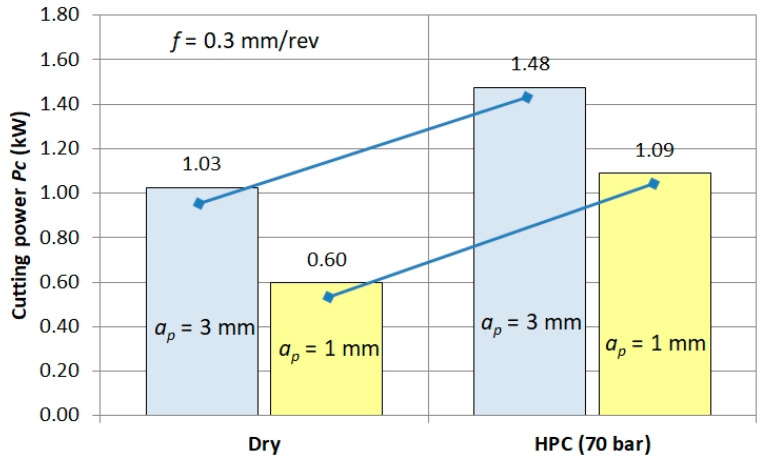
Example of *Pc* power demand for dry machining and HPC.

An exemplary combination of *Pc* power for dry machining and HPC is shown in Figure 12. It follows that the power demand for HPC machining is greater by about *Pc* ≈ 0.5 kW. This also confirms the thesis that, due to the form of chips, the application of the machining process at elevated pressure should be reduced.

In connection with the above, a case study was carried out, for which the algorithm shown in Figure 4 was used. It was assumed that the workpiece was a stepped shaft with external dimensions Ø50 × 80 mm. The cutting depth for roughing operations is *a_p_* = 3.0 mm and for finishing *a_p_* = 1.0 mm. The number of passes for rough turning *n* = 11 and one pass for finishing turning along the contour were assumed.

**Figure 13:**
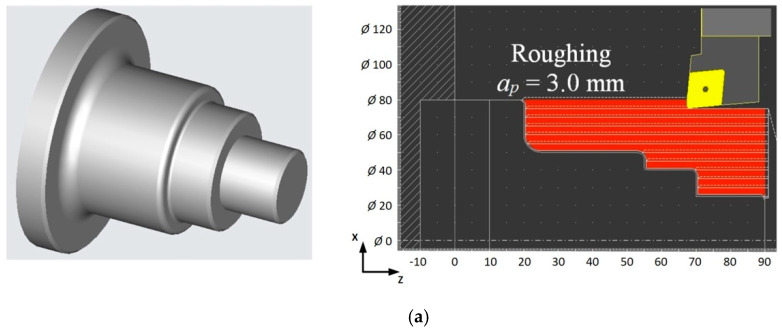

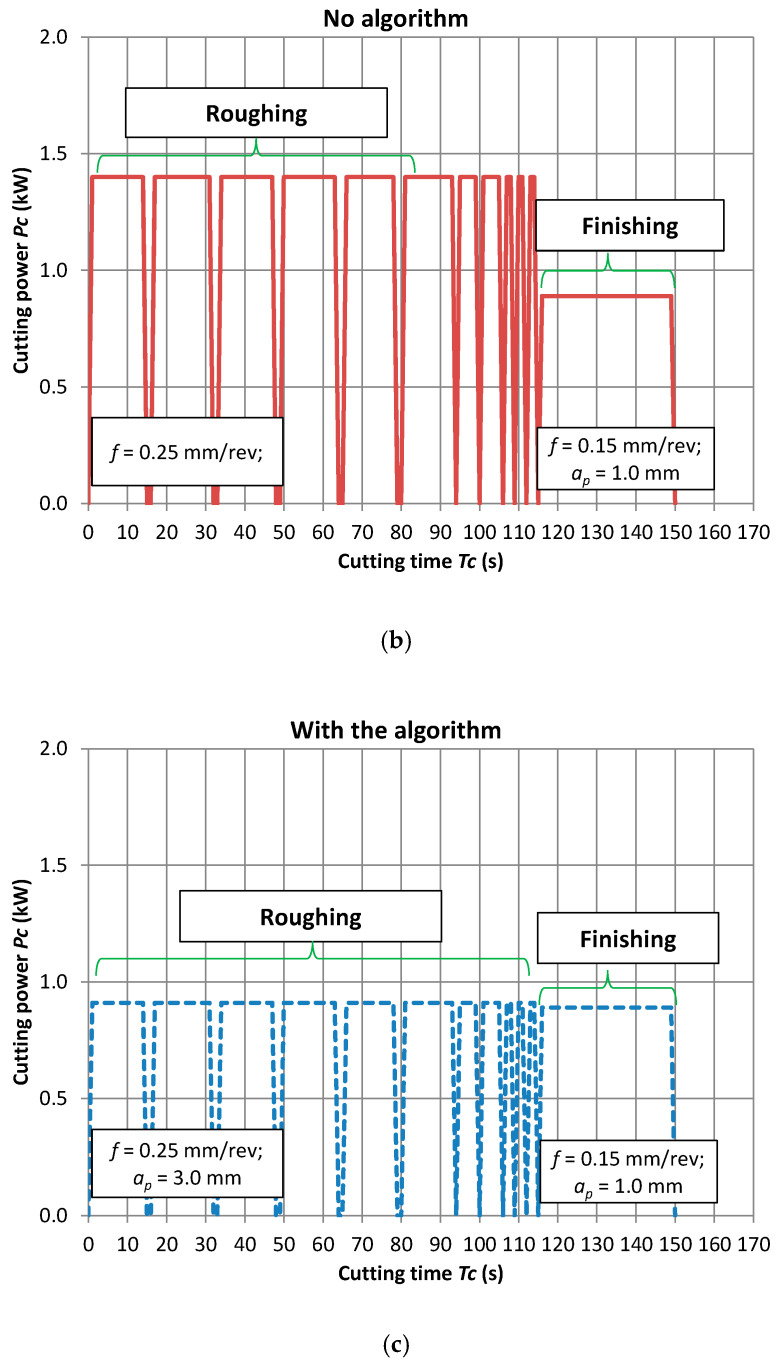
Example illustrating the operation of the machining optimization algorithm (**a**) view of parts and machining paths, (**b**) power demand course during cutting time (*Tc*) without the algorithm, (**c**) power demand course during cutting time (*Tc*) with the algorithm used.

Figure 13a shows a view of the workpiece and the cutting tool path during roughing turning. Figure 13b shows the diagram of power demand for the case of HPC turning throughout the entire cutting time (*Tc*). Figure 13c depicts the diagram of power demand for the case of HPC turning with the implementation of the algorithm. As a result, the cutting power for rough turning was reduced, while ensuring the required chip geometry.

Approximate CO_2_ emission for dry machining and HPC was estimated in accordance with formulas (2) to (4). The following assumptions were made for the calculations:

- Value of the emission factor for electricity of the final customers in Poland in 2019, *CEF_elec_* = 765 kg/MWh (data based on KOBiZE [49]);
- *Pc* value for roughing based on the case study: *Pc* = 0.91 kW;
- *Pa* = 0.2∙*Pc* = 0.182 kW (the value of the coefficient *b_m_* = 0.2 was adopted based on Liu et al. [47]);
- Machine tool power consumption when idle *P_air_* = 1.0 kW;
- *P_add system_* = 0.5 kW – 40% of the maximum load of the machine tool pump feeding the coolant was taken for calculations;
- Total roughing time in one business day *T_c roughing_* = 8 h.

On the basis of the adopted values, the energy consumption and carbon emission were calculated, i.e., *EC_process dry_* = 2.1 kW and *EC_process HPC_* = 2.6 kW. This means that the value of carbon emissions per year is: *CE_elec dry_* = 3083 kgCO_2_/year and *CE_elec HPC_* = 3817 kgCO_2_/year. It follows that, for such assumptions, the difference in carbon emissions within one year is *CE_elec HPC_ − CE_elec dry_* = 734 kgCO_2_.

## 4. Summary and Conclusions

An algorithm (Figure 5) for the optimization of the cutting process due to the chip’s geometry obtained during machining of titanium alloy was elaborated. A simplified method of determining the coefficient indicating the geometry and chip breaking, which can be used in industrial practice, was developed and presented.

On the basis of the obtained results and the performed analysis, the following conclusions can be drawn. Obtaining the appropriate chip geometry in the turning process with elevated coolant pressure can be achieved with reduced power requirements. This is due to the selected cutting parameters values, i.e., the feed *f* and cutting depth *a_p_*, for which the chip breaker groove is correctly filled. This translates into improved chip breaking process and the creation of the correct/acceptable chip geometry. In the analyzed case, it was shown that the correct chip geometry can be obtained with a reduced power requirement by approximately *Pc* = 0.5 kW. It was estimated that, for the analyzed case, the CO_2_ savings during the year was approximately 734 kgCO_2_.

It has been proven that there is no significant influence of HPC turning on the workpiece surface roughness. However, for finishing, the recommended machining method should implement HPC system, taking into account chip geometry.

Although in the presented case the saving is obvious, there is a need to continue testing the algorithm taking into account the wear process of the cutting edge. It can have significant impact on chip geometry. In addition, the limitation is the scope of research covering one type, although the most popular in industrial applications, of titanium alloy i.e., Ti6Al4V. This issue requires further research and analysis in the field of turning difficult to cut materials.

## Figures and Tables

**Table 1:** Mechanical properties of the material Ti6Al4V.

Material	Tensile Strength Rm (MPa)	Elongation A_5_ (%)	Rockwell Hardness HRC	Density (g/mm^3^)
Ti6Al4V	1020	14	33	4.44

**Table 2:** Chemical composition of Ti6Al4V (%).

Ti	V	Al	Fe	Cu	H	O	N
rest	4	6	0.1	0.03	0.03	0.15	0.01

**Table 3:** Research plan with real values.

No.	A	B	C	*f*	*a_p_*	*p*
				(mm/rev)	(mm)	(bar)
1	1	1	1	0.15	1	0
2	1	1	1	0.15	1	0
3	1	2	2	0.15	3	70
4	1	2	2	0.15	3	70
5	2	1	1	0.20	1	0
6	2	1	1	0.20	1	0
7	2	2	2	0.20	3	70
8	2	2	2	0.20	3	70
9	3	1	2	0.25	1	70
10	3	1	2	0.25	1	70
11	3	2	1	0.25	3	0
12	3	2	1	0.25	3	0
13	4	1	2	0.30	1	70
14	4	1	2	0.30	1	70
15	4	2	1	0.30	3	0
16	4	2	1	0.30	3	0

A, B, C—factors in Taguchi array design.

**Table 4:** Test results for cutting forces measurements *F_c_*, *F_f_*, *F_p_*.

No.	*f*	*a_p_*	*p*	S/N *F_c_*	*F_c mean_*	S/N *F_f_*	*F_f mean_*	S/N *F_p_*	*F_p mean_*
	(mm/rev)	(mm)	(bar)		(N)		(N)		(N)
1	0.15	1	0	−50.5	334.8	−41.7	120.8	−41.0	111.2
2	0.15	1	0						
3	0.15	3	70	−57.1	716.0	−53.1	450.5	−42.0	126.1
4	0.15	3	70						
5	0.20	1	0	−52.4	415.7	−42.2	128.0	−41.6	118.7
6	0.20	1	0						
7	0.20	3	70	−57.9	787.4	−54.3	517.1	−43.5	149.3
8	0.20	3	70						
9	0.25	1	70	−53.2	459.0	−42.1	126.8	−41.1	113.6
10	0.25	1	70						
11	0.25	3	0	−59.0	892.5	−54.6	535.8	−45.2	181.6
12	0.25	3	0						
13	0.30	1	70	−55.3	579.0	−46.6	213.9	−45.0	178.2
14	0.30	1	70						
15	0.30	3	0	-60.0	1004.4	-55.6	600.8	-46.7	215.2
16	0.30	3	0						

**Table 5:** Analysis of variance for mean values—main force *F_c._*

Source	*DF*	*Seq SS*	*Adj SS*	*Adj MS*	*F*	*P*
*f* (mm/rev)	3	77,199	77,199	25,733	1320.27	0.001
*a_p_* (mm)	1	324,766	324,766	324,766	16,662.60	0.000
*p* (bar)	1	1399	1399	1399	71.80	0.014
Residual Error	2	39	39	19		
Total	7	403,404				

**Table 6:** Analysis of variance for mean values—feed force *F_f._*

Source	*DF*	*Seq SS*	*Adj SS*	*Adj MS*	*F*	*P*
*f* (mm/rev)	3	15,646	15,646	5215	10.40	0.089
*a_p_* (mm)	1	286,815	286,815	286,815	571.84	0.002
*p* (bar)	1	743	743	743	1.48	0.34
Residual Error	2	1003	1003	502		
Total	7	304,207				

**Table 7:** Analysis of variance for mean values—thrust force *F_p._*

Source	*DF*	*Seq SS*	*Adj SS*	*Adj MS*	*F*	*P*
*f* (mm/rev)	3	6842.8	6842.8	2280.9	15.18	0.062
*a_p_* (mm)	1	2833.2	2833.2	2833.2	18.86	0.049
*p* (bar)	1	443.5	443.5	443.5	2.95	0.228
Residual Error	2	300.5	300.5	150.2		
Total	7	10,420.0				

**Table 8:** Average power consumption (*Pc*), chip length (*Lch*) and chip breaking index (*C_in_*) values for dry machining.

Dry Machining					
No.	*f*	*a_p_*	*Pc*	*Lch*	*C_in_*
	(mm/rev)	(mm)	(kW)	(mm)	
1	0.15	1	0.34	37.5	0.75
2	0.15	3	0.75	22.3	0.45
3	0.2	1	0.42	63.8	1.00
4	0.2	3	0.82	33.5	0.67
5	0.25	1	0.48	22.0	0.44
6	0.25	3	0.91	13.0	0.26
7	0.3	1	0.60	14.0	0.28
8	0.3	3	1.03	11.0	0.22

**Table 9:** Average *Pc*, *Lch* and *C_in_* values for HPC treatment.

HPC Machining (70 bar)					
No.	*f*	*a_p_*	*Pc*	*Lch*	*C_in_*
	(mm/rev)	(mm)	(kW)	(mm)	
1	0.15	1	0.80	2.5	0.05
2	0.15	3	1.23	6.0	0.12
3	0.2	1	0.90	2.9	0.06
4	0.2	3	1.30	7.1	0.14
5	0.25	1	0.97	3.3	0.07
6	0.25	3	1.40	9.0	0.18
7	0.3	1	1.09	3.8	0.08
8	0.3	3	1.48	12.0	0.24

## Nomenclature

*f*	Feed rate	*Pc*	Cutting Power
*a_p_*	Depth of cut	*F_c_*	Main component of the total cutting force
*r_Ɛ_*	Nose radius	*F_f_*	Feed component of the total cutting force
*v_c_*	Cutting speed	*Fp*	Radial component of the total cutting force
*CE*	Carbon emission by CNC machining system	*Lch*	Chip length
*C_in_*	Chip breakability index	*Tc*	Cutting time
*EC*	Energy Consumption	*p*	Pressure
*EC*_process_	Energy consumption of the CNC machine tool	*Ra*	Arithmetical mean deviation of the assessed profile
